# Alkaline Phosphatase, Soluble Extracellular Adenine Nucleotides, and Adenosine Production after Infant Cardiopulmonary Bypass

**DOI:** 10.1371/journal.pone.0158981

**Published:** 2016-07-06

**Authors:** Jesse A. Davidson, Tracy Urban, Suhong Tong, Mark Twite, Alan Woodruff, Paul E. Wischmeyer, Jelena Klawitter

**Affiliations:** 1 Department of Pediatrics, Pediatric Cardiology, University of Colorado, Aurora, CO, United States of America; 2 Children’s Hospital Colorado, CCRO, Aurora, CO, United States of America; 3 Department of Biostatistics, University of Colorado, Aurora, CO, United States of America; 4 Department of Anesthesiology, University of Colorado, Aurora, CO, United States of America; 5 Division of Pediatric Critical Care Medicine, Boston Children’s Hospital, Department of Anesthesia/Harvard Medical School, Boston, MA, United States of America; CCAC, UNITED STATES

## Abstract

**Rationale:**

Decreased alkaline phosphatase activity after infant cardiac surgery is associated with increased post-operative cardiovascular support requirements. In adults undergoing coronary artery bypass grafting, alkaline phosphatase infusion may reduce inflammation. Mechanisms underlying these effects have not been explored but may include decreased conversion of extracellular adenine nucleotides to adenosine.

**Objectives:**

1) Evaluate the association between alkaline phosphatase activity and serum conversion of adenosine monophosphate to adenosine after infant cardiac surgery; 2) assess if inhibition/supplementation of serum alkaline phosphatase modulates this conversion.

**Methods and Research:**

Pre/post-bypass serum samples were obtained from 75 infants <4 months of age. Serum conversion of 13C5-adenosine monophosphate to 13C5-adenosine was assessed with/without selective inhibition of alkaline phosphatase and CD73. Low and high concentration 13C5-adenosine monophosphate (simulating normal/stress concentrations) were used. Effects of alkaline phosphatase supplementation on adenosine monophosphate clearance were also assessed. Changes in serum alkaline phosphatase activity were strongly correlated with changes in 13C5-adenosine production with or without CD73 inhibition (r = 0.83; p<0.0001). Serum with low alkaline phosphatase activity (≤80 U/L) generated significantly less 13C5-adenosine, particularly in the presence of high concentration 13C5-adenosine monophosphate (10.4μmol/L vs 12.9μmol/L; p = 0.0004). Inhibition of alkaline phosphatase led to a marked decrease in 13C5-adenosine production (11.9μmol/L vs 2.7μmol/L; p<0.0001). Supplementation with physiologic dose human tissue non-specific alkaline phosphatase or high dose bovine intestinal alkaline phosphatase doubled 13C5-adenosine monophosphate conversion to 13C5-adenosine (p<0.0001).

**Conclusions:**

Alkaline phosphatase represents the primary serum ectonucleotidase after infant cardiac surgery and low post-operative alkaline phosphatase activity leads to impaired capacity to clear adenosine monophosphate. AP supplementation improves serum clearance of adenosine monophosphate to adenosine. These findings represent a potential therapeutic mechanism for alkaline phosphatase infusion during cardiac surgery.

**New and Noteworthy:**

We identify alkaline phosphatase (AP) as the primary soluble ectonucleotidase in infants undergoing cardiopulmonary bypass and show decreased capacity to clear AMP when AP activity decreases post-bypass. Supplementation of AP ex vivo improves this capacity and may represent the beneficial therapeutic mechanism of AP infusion seen in phase 2 studies.

## Introduction

Congenital cardiovascular defects are a significant cause of morbidity and mortality in children. In the United States alone an estimated 189,000 life years are lost before age 55 due to congenital cardiovascular defects, comparable to leukemia and asthma combined.[1] Survivors are often subject to significant morbidities including chronic heart failure, renal failure, hepatic dysfunction, intestinal injury, and neurologic injury.[1–5]

Cardiothoracic surgery with cardiopulmonary bypass (CPB) is often necessary as a life-saving procedure in children with congenital heart disease. Unfortunately, surgery and CPB themselves routinely result in multiple systemic derangements including global ischemia/reperfusion [6], the systemic inflammatory response syndrome [7,8], and organ dysfunction including low cardiac output syndrome.[9] Infants with complex congenital heart lesions have a particularly high risk of injury during the perioperative period and new therapies are needed to decrease acute and chronic morbidity in this population.

Recent evidence points to a potential role for alkaline phosphatase (AP) as part of the host defense system against inflammation and tissue injury in multiple disease processes.[10–18] Alkaline phosphatases are a family of endogenous metalloenzymes present in serum and most organs.[19] Infants undergoing cardiac surgery with CPB demonstrate a precipitous post-operative decline in serum AP activity, and low activity on post-operative day one is associated with increased inflammation and increased cardiovascular support requirements.[20] Adult cardiac surgery patients demonstrate a similar decline in AP activity, and a pilot study of continuous AP infusion during coronary artery bypass grafting led to a significant decrease in systemic inflammation.[21,22]

The potential therapeutic mechanisms of AP in this setting are unclear. All AP’s are capable of hydrolytic phosphatase activity targeting a variety of molecules. Recently, specific targets of AP related to human health and disease have been described. Perhaps the most intriguing targets are extracellular adenine nucleotides released through cellular apoptosis or necrosis during ischemia/reperfusion injury. Extracellular adenosine triphosphate (ATP), adenosine diphosphate (ADP), and adenosine monophosphate (AMP) lead to inflammatory activation, vasoconstriction, and platelet activation while stepwise dephosphorylation of these substrates to adenosine may be protective.[23–25] Ecto-5’-nucleotidase (CD73) has been hypothesized to be the primary enzyme capable of dephosphorylating extracellular AMP to adenosine. [26] However, in healthy neonates, soluble AP has been shown to have a similar capacity to convert AMP to adenosine.[26]

Given this function of AP in the healthy neonate, we hypothesized that the decrease in AP activity routinely seen after infant cardiothoracic surgery would lead to a reduced serum capacity to dephosphorylate extracellular AMP. We also sought to demonstrate that AP continues to be the primary serum ectonucleotidase in this pathologic setting and that serum capacity to convert AMP to adenosine would correlate with residual AP activity. Together, these results would help identify a key mechanism linking low AP activity to inflammation and increased cardiovascular support requirements after infant cardiothoracic surgery. In addition, while AP therapy has demonstrated promising early results in multiple diseases [22,27–32], the effects of therapeutic AP administration on adenine nucleotide clearance and adenosine production are unknown. Therefore, we explored the ex vivo effects of exogenous AP supplementation on AMP dephosphorylation to evaluate if AP modulates this pathway as part of its therapeutic mechanism.

## Materials and Methods

### Study Design and Participants

This study was performed as a primary aim of a prospective cohort study assessing the biology and kinetics of AP in infants undergoing surgical repair or palliation of congenital heart disease. All surgeries took place at Children’s Hospital Colorado between September 2013 and January 2015. Inclusion criteria were age ≤120 days at the time of surgery and the use of CPB for repair. Patients were excluded if they were less than 34 weeks corrected gestational age or weighed less than 2kg at the time of surgery in order to avoid excessive research blood draws and risk of anemia. The study was approved by the Colorado Multiple Institutional Review Board and performed in accordance with the institutional guidelines of Children’s Hospital Colorado and the University of Colorado. Written consent was obtained from parents in all cases, as all subjects were infants and below the consent/assent age.

### Sample Collection and Processing

Serum samples were obtained from indwelling arterial or venous catheters after induction of anesthesia and prior to first surgical incision (“pre-operative”). A portion of the sample was frozen at -20°C and shipped to Mayo Medical Laboratories for assessment of serum AP activity while the remaining serum was stored frozen at -70°C for batch analysis of AMP to adenosine conversion. A second serum sample was obtained from each subject during the rewarming phase of CPB with identical processing (“rewarming”). We limited sampling to the pre-bypass and rewarming periods in order to minimize the risk of post-operative anemia in this critically ill patient population.

### Alkaline Phosphatase Activity Assays

AP activity was determined on each sample using a clinically available photometric p-nitrophenol phosphate cleavage assay (Mayo Medical Laboratories, Rochester MN). Briefly, AP cleaves p-nitrophenol phosphate in the presence of magnesium to yield phosphate and n-nitrophenol. The rate of p-nitrophenol production (determined photometrically at 450 nm) is directly proportional to AP activity. Based on our prior study, low AP activity in this population was defined a priori as ≤80 U/L.[20]

### AMP/Adenosine Conversion Assays

Stored serum was assessed ex vivo via HPLC-MS/MS for conversion of exogenous 13C5-AMP to 13C5-adenosine (both Toronto Research Chemicals, Toronto, ON, Canada). Low (5 μmol/L; patients 1–25) and high (50 μmol/L; patients 26–50) concentration 13C5-AMP were used to simulate normal/stress concentrations of AMP.[26] Reactions were terminated after 15 minutes by addition of five volumes acetonitrile/ methanol containing 5 μmol/L d1-adenosine as internal standard. In all assays, adenosine deaminase 1 (ADA1) and equilibrative nucleoside transporter 1 and 2 (ENT1,2) were blocked with erythro-9-(2-hydroxy-3-nonyl)adenine (EHNA) and dipyridamole respectively [Tocris Cookson; Bristol, UK] in order to prevent adenosine breakdown/reuptake respectively, thus trapping the adenosine allowing accurate measurement of its production.[26] Native AMP and adenosine levels were also measured to assess background signal. For more detail, please refer to the supplementary methods section (S1 File).

### Alkaline Phosphatase Inhibition/Supplementation

We next assessed ex vivo 13C-AMP conversion to adenosine with and without selective inhibition of tissue non-specific AP (TNAP) and CD73, the other major soluble ectonucleotidase. TNAP inhibition was accomplished using MLS-0038949 [EMD Millipore (Billerica, MA, USA)], and CD73 inhibition through addition of adenosine 5’-(α,β-methylene)-diphosphate [Sigma (St. Louis, MO, USA)].[26]

The final set of assays sought to determine if addition of exogenous AP could increase 13C5-AMP clearance to 13C5-adenosine in the presence of high concentrations of 13C5-AMP (50μmol/L). AP was tested in two forms: bovine intestinal AP (BiAP) (Alloksys Life Sciences B.V., Bunnik, NL) and human liver AP (MyBioSource, San Diego, CA). BiAP is the primary form used for therapeutic trials in both intestinal and systemic disease, making testing of this potential therapeutic mechanism clinically relevant.[22,30–32] We chose to also test human liver AP in this setting to evaluate if additive benefit could be derived by supplementing with one of the isoforms most abundant in serum under typical conditions. Five dosing strategies were chosen: no supplementation, low dose BiAP (500 U/L), low dose BiAP + low dose liver AP (both at concentrations of 500 U/L), high dose BiAP (50,000 U/L), and high dose BiAP + low dose liver AP. ADA1 and ENT1/2 were inhibited in all samples. CD73 was not inhibited for these assays as we sought to demonstrate increased adenosine production above the total baseline serum ectonucleotidase capacity.

### Statistical Analysis

Patients’ demographics and baseline clinic characteristics were summarized using mean and standard deviation for continuous variables, while frequency and percent were used for dichotomized variables. Two-sample t-test, Chi-square/Fisher’s exact test as appropriate were performed to compare the difference between groups. Pearson’s correlation test was performed to evaluate the correlation between AP total activity and adenosine product at pre-operation and pre-separation time points. Paired t-test was performed to compare the ratio of 13C5-adenosine production with and without CD73 inhibition. ANOVA method and paired t-tests were employed to compare adenosine production by various AP supplementation strategies. All the data analyses were performed using SAS V9.4, and graphs were polished in GraphPad Prism 6.0 using the output data from SAS.

## Results

### Subjects

The first 76 subjects enrolled in the cohort were included in this arm of the study. One was excluded from the analysis due to insufficient serum sample volume. Baseline characteristics are presented in Table 1. Our cohort was typical for the overall infant population in our system, demonstrating a high percentage for neonates with critical heart disease, high comprehensive Aristotle scores, and one quarter of patients exhibiting single ventricle physiology. A quarter of patients required mechanical ventilation at some stage during the pre-operative period and 12% required pre-operative inotropic support. Surgical variables included a wide range of CPB and aortic cross clamp times with relatively frequent use of either deep hypothermic circulatory arrest or selective cerebral perfusion.

**Table 1:** Clinical characteristics.

	Cohort	Rewarming APActivity ≤80U/L	Rewarming AP Activity >80U/L N = 49	p-value ≤80 vs >80
	N = 75	N = 24		
Male (%)	43 (57.3%)	14 (58.3%)	27 (55.1%)	0.79
Age at surgery, days; median[range]	18 (1, 119)	4.5 (1, 108)	46 (4, 119)	<0.0001
Weight at surgery, kg; median[range]	3.5 (2.2, 7.2)	3.1 (2.2, 4.9)	4.0 (2.3, 7.2)	<0.001
Pre-operation intubation (%)	20 (27.4%)	8 (33.3%)	11 (23.4%)	0.37
Pre-operation inotropic/vasoactive support (%)	9 (12.0%)	3 (12.5%)	6 (12.2%)	1.0
Pre-operation steroids (%)	40 (55.6%)	19 (79.2%)	20 (43.5%)	<0.005
Single ventricle physiology (%)	18 (24.0%)	8 (33.3%)	9 (18.4%)	0.15
Aristotle Score-Comprehensive; median[range]	8.5 (3.0, 15.0)	10 (3, 15)	8 (3, 14.5)	< 0.05
Cardiopulmonary bypass time, minutes; median[range]	122 (54, 399)	148.5 (75, 399)	119 (54, 215)	<0.01
Cross-clamp time, minutes; median[range]	69 (0, 241)	80 (0, 241)	62 (0, 142)	<0.005
Deep hypothermic circulatory arrest, minutes; median[range]	0 (0, 77)	7.5 (0, 77)	0 (0, 59)	0.001
Selective cerebral perfusion, minutes; media[range]	0 (0, 115)	0 (0, 115)	0 (0, 37)	0.12

### AP Activity

A total of 72 subjects had AP activity analyzed in both the pre-operative and rewarming samples. AP activity could not be determined in one pre-operative sample and two rewarming samples due to interference from gross hemolysis. Mean total AP activity in the cohort decreased from 199.5 U/L pre-operation to 103.9 U/L during rewarming (p<0.0001). Pre-operative AP activity was low in three subjects (4%) based on the a priori definition of AP activity ≤80 U/L. At the rewarming stage, this number increased to 24 subjects (33%).

### AP Activity versus Adenosine Production

To assess the importance of total AP activity for determining serum capacity to clear AMP to adenosine, we first examined the correlation of AP activity to 13C5-adenosine production under different experimental conditions. These results are shown in Table 2. Overall, total AP activity showed a strong correlation with 13C5-adenosine production at the pre-operative time point. The correlation was higher in the high concentration 13C5-AMP group compared to the low concentration 13C5-AMP group suggesting that AP activity may be a more important determinant of AMP clearance at higher concentrations of AMP. CD73 inhibition increased the correlation of AP activity with 13C5-adenosine production at low concentrations of 13C5-AMP but did not change the correlation when the higher concentration 13C5-AMP was used.

**Table 2:** Correlation of AP activity to 13C5-adenosine production pre-operation and prior to separation from cardiopulmonary bypass.

	Pre-operative	Rewarming
	r (p-value)	
**Low concentration 13C-AMP**		
Without CD73 Inhibition	0.70 (< 0.0001)	0.48 (<0.05)
With CD73 Inhibition	0.82 (< 0.0001)	0.80 (< 0.0001)
**High concentration 13C-AMP**		
Without CD73 Inhibition	0.87 (< 0.0001)	0.76 (< 0.0001)
With CD73 Inhibition	0.88 (< 0.0001)	0.70 (0.0001)

During rewarming the correlation between AP activity and 13C5-adenosine production decreased (Table 2). This decrease was most profound in the low concentration 13C5-AMP group without CD73 inhibition, but the decrease was present under all conditions.

We also tested our hypothesis that rewarming serum samples with low AP activity (≤80 U/L) would demonstrate decreased capacity to clear 13C5-AMP to 13C5-adenosine. Post-operative serum from infants with low AP activity generated significantly less 13C5-adenosine when challenged with high concentration 13C5-AMP (12.9 μmol/L vs 10.4μmol/L; p<0.0005). A similar trend was present that did not reach statistical significance for rewarming serum challenged with low concentration 13C5-AMP (2.0 μmol/L vs 1.9 μmol/L; p = 0.13). Comparing samples in the upper and lower quartiles of AP activity, we found a larger difference in adenosine production when challenged with high concentration 13C5-AMP (14.2 μmol/L vs 9.7 μmol/L; p<0.005) and a similar trend with low concentration 13C5-AMP (2.1 μmol/L vs 1.8 μmol/L; p = 0.08). When CD73 was inhibited, serum from infants with low AP activity produced significantly less 13C5-adenosine when challenged with either high or low concentration 13C5-AMP (8.7 μmol/L vs 7.7 μmol/L; p<0.05 and 1.4 μmol/L vs 1.1 μmol/L; p<0.005 respectively). Again these differences were more marked when the lowest and highest quartiles were compared (10.1 μmol/L vs 7.5 μmol/L; p<0.005 and 1.6 μmol/L vs 1.1 μmol/L; p<0.01 respectively).

### Change in AP Activity versus Change in Adenosine Production

Next we evaluated if decreased AP activity across time points (pre-operative to rewarming) correlated directly with the change in serum capacity to convert AMP to adenosine. In serum challenged with low concentration 13C5-AMP, we found a statistically significant correlation between change in AP activity and change in 13C5-adenosine production (r = 0.50; p<0.05). This correlation increased modestly with addition of CD73 inhibition (r = 0.57; p<0.005). In serum challenged with high concentration 13C5-AMP, the correlation between change in AP activity and change in 13C5-adenosine production was much stronger with (r = 0.84; p<0.0001) or without (r = 0.83; p<0.0001) CD73 inhibition.

**Fig 1:**
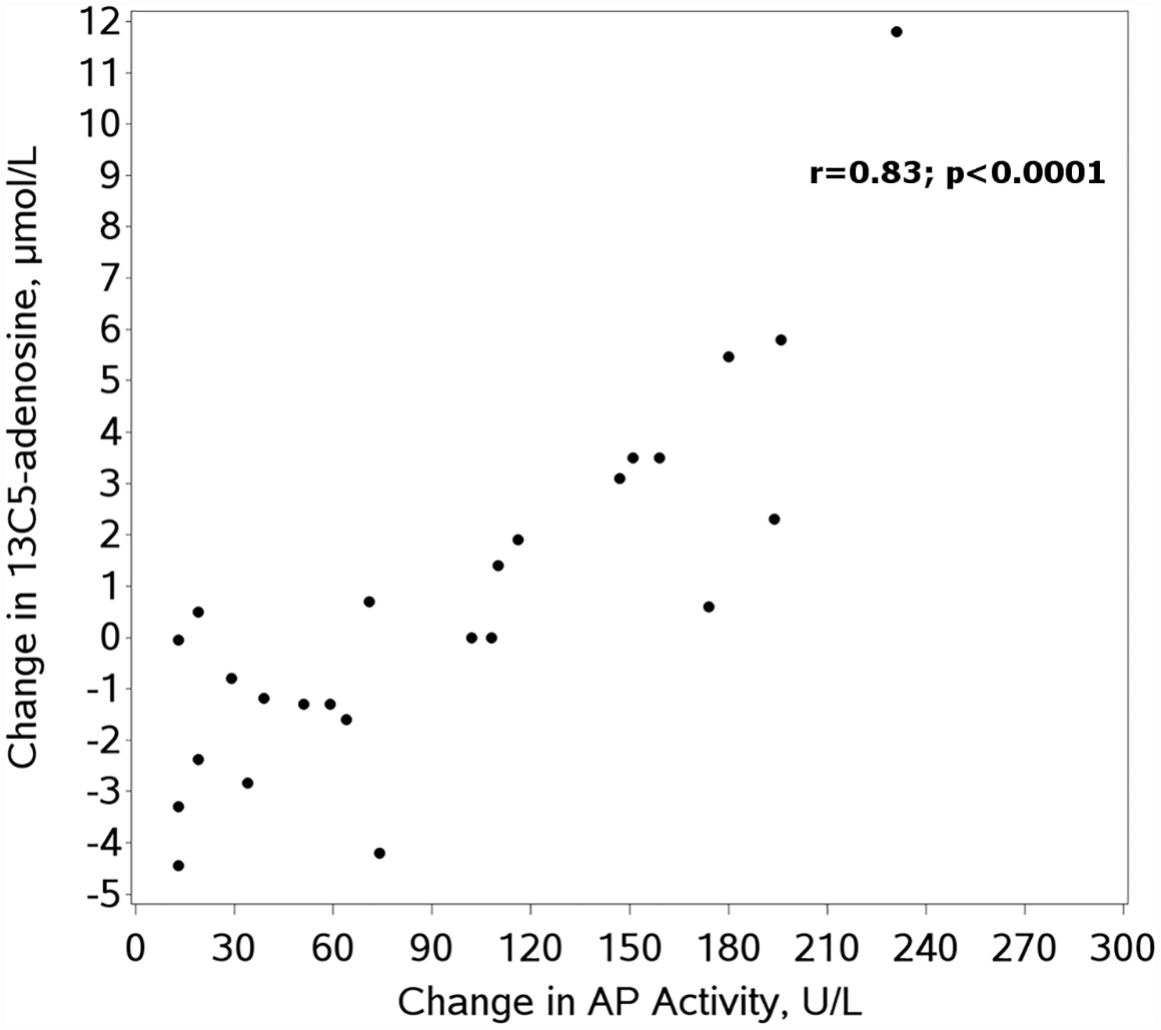
Pre-operation to rewarming change in alkaline phosphatase activity versus change in 13C5-adenosine without CD73 inhibition. AP = alkaline phosphatase.

**Fig 2:**
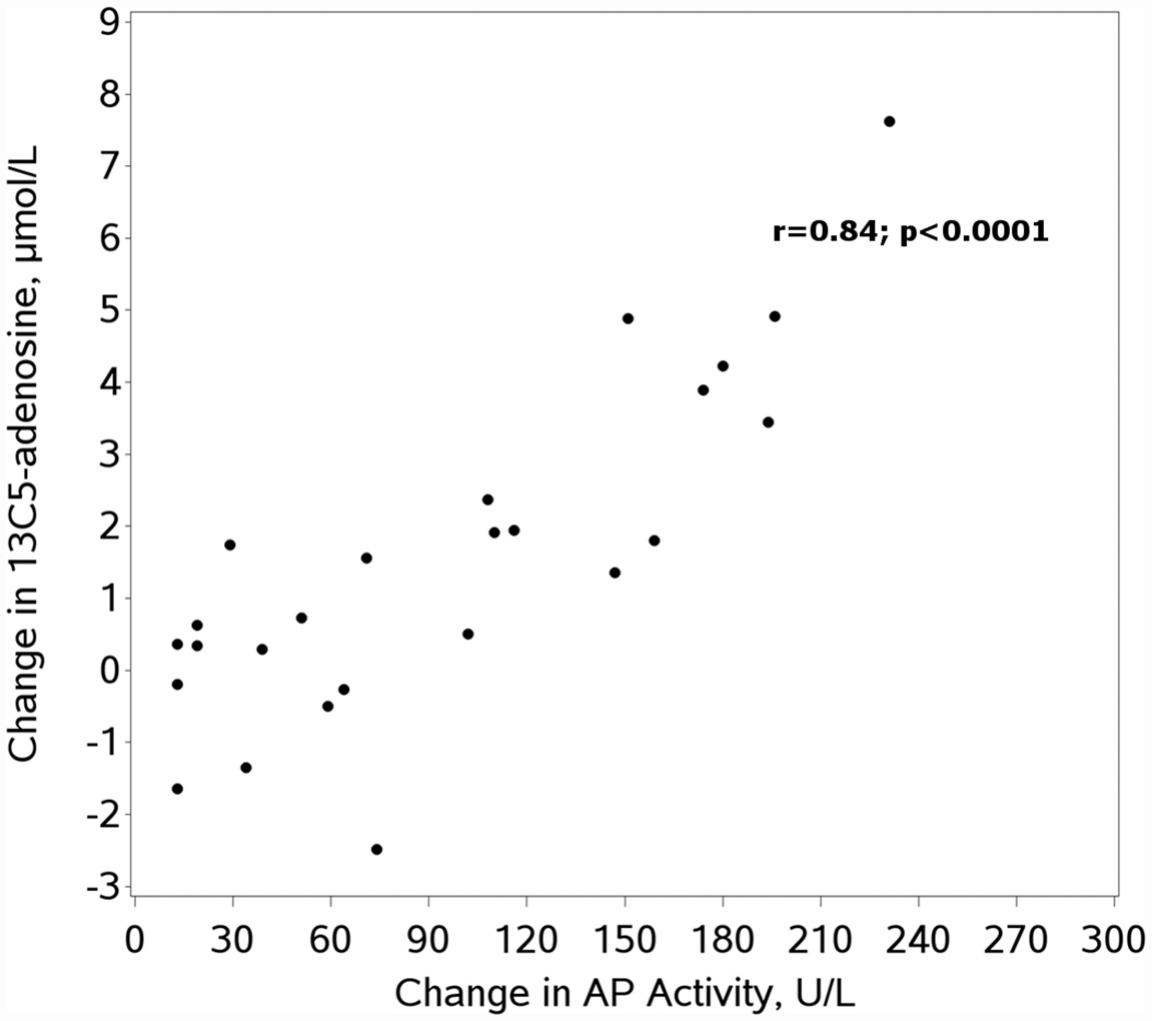
Pre-operation to rewarming change in alkaline phosphatase activity versus change in 13C5-adenosine with CD73 inhibition. AP = alkaline phosphatase.

Interestingly, while changes in AP activity appeared to be the primary driver of changes in 13C5-adenosine, 13C5-adenosine production did not decrease in all subjects during rewarming despite decreases in AP activity in all subjects (Fig 1). In subjects with minimal change in AP activity between the pre-operative and rewarming time points, we observed a rise in 13C5-adenosine production at the post-operative time point, which was substantially blunted by inhibition of CD73 (Fig 2). CD73 inhibition did not, however, appear to change the overall association of AP activity and 13C5-adenosine production, suggesting a relatively constant effect of CD73 across all rewarming samples. While limits on the volume of blood drawn for research prohibited us from directly assessing this unexpected finding of potentially increased CD73 activity post-operatively, we did indirectly assess the importance of CD73 through comparison of the ratio of 13C5-adenosine production with and without CD73 inhibition in pre-operative and rewarming samples. These results are shown in Table 3. Across the entire cohort, this ratio decreased substantially post-operatively, indicating a more prominent role for CD73 post-operatively. Also, CD73 appeared to play a more substantial role at lower concentrations of 13C5-AMP with a less prominent effect at high 13C5-AMP concentrations.

**Table 3:** Ratio of 13C5-adenosine production with and without CD73 inhibition, pre-operation and prior to separation from cardiopulmonary bypass.

	Pre-operative, mean [SD]	Rewarming, mean [SD]	Pre-operative to Rewarming Mean Difference (95% CI)	p-value
**Entire Cohort**	0.75 (0.09)	0.68 (0.08)	0.08 (0.05, 0.11)	<0.0001
**Low Concentration 13C5-AMP**	0.72 (0.10)	0.65 (0.09)	0.07 (0.03, 0.10)	<0.005
**High Concentration 13C5-AMP**	0.79 (0.07)	0.70 (0.07)	0.09 (0.04, 0.13)	<0.001

### AP Inhibition

**Fig 3:**
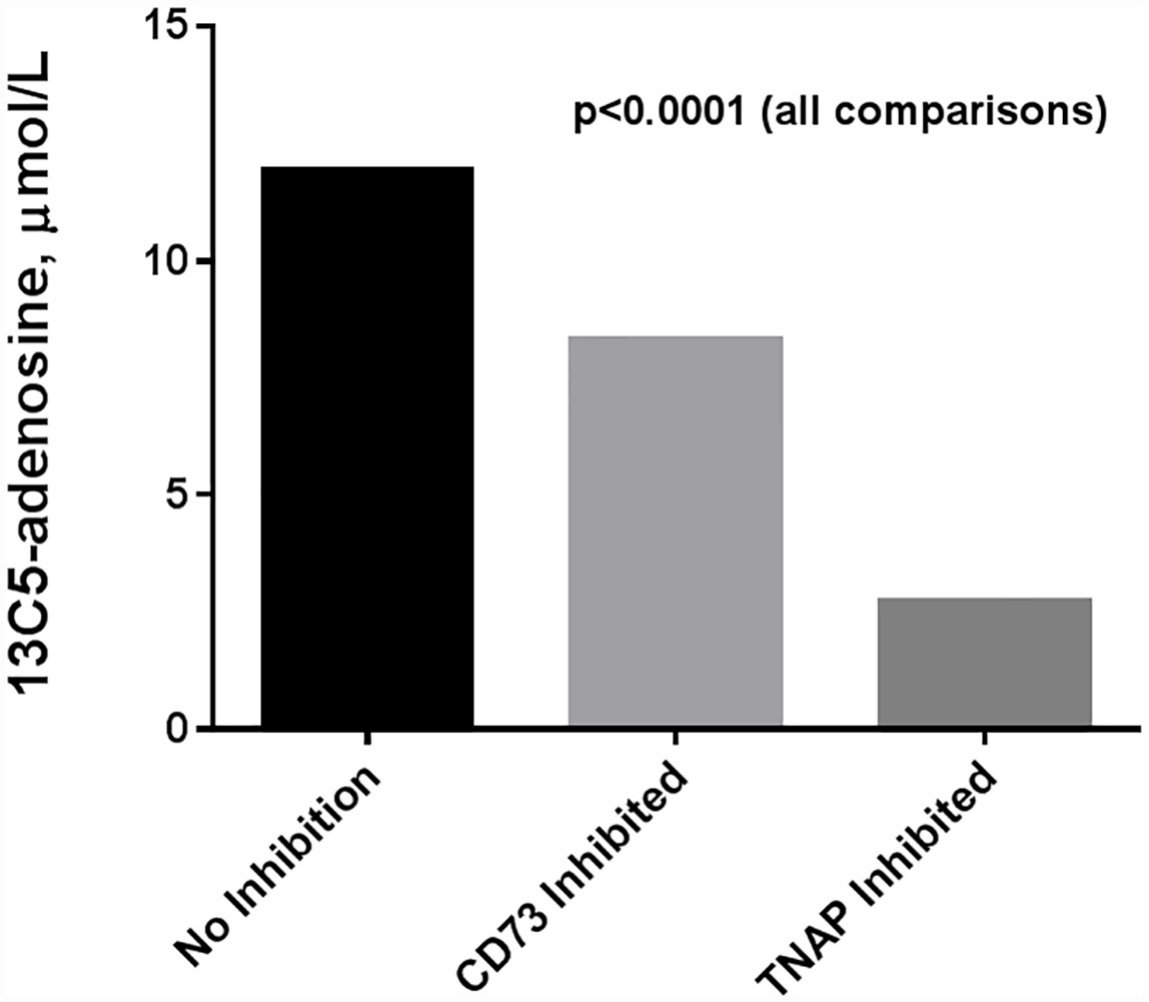
13C5-adenosine production without ectonucleotidase inhibition, with CD73 inhibition, and with alkaline phosphatase inhibition. TNAP = tissue non-specific alkaline phosphatase.

The strong correlation of serum AP activity to serum 13C5-adenosine production is highly suggestive that AP is the primary serum ectonucleotidase in our patient population. To directly test the contribution of AP to adenosine production, we used a known selective inhibitor of TNAP (MLS-0038949) to block AP activity in our post-operative samples from subjects 26–50 challenged with high concentration 13C5-AMP (Fig 3). Inhibition of TNAP led to a marked decrease in 13C5-adenosine production (11.9 μmol/L vs 2.7 μmol/L; p<0.0001). In comparison, selective inhibition of CD73 was significantly less effective than TNAP inhibition for blocking 13C5-adenosine production (8.3 μmol/L vs 2.7 μmol/L; p<0.0001).

### AP Supplementation

On average only 40% of 13C5-AMP added at low concentration and 25% of 13C5-AMP added at high concentration was converted to 13C5-adenosine in serum from the first 50 subjects, leaving a substantial concentration of 13C5-AMP in the serum at the time of reaction termination. One of the proposed beneficial mechanisms of AP therapy in disease is increased clearance of AMP and other extracellular adenine nucleotides to adenosine.[26,33–34] Therefore as a final assay, we explored the effects of the addition of exogenous AP on serum capacity to clear 13C5-AMP to adenosine in samples collected from the final 25 subjects. Initial testing was performed using BiAP, as this is the formulation currently under evaluation in therapeutic trials for sepsis, ulcerative colitis, and cardiac surgery. Subsequently we added human liver AP, one of the isoforms typically present in human serum, to assess for additive effects.

**Fig 4:**
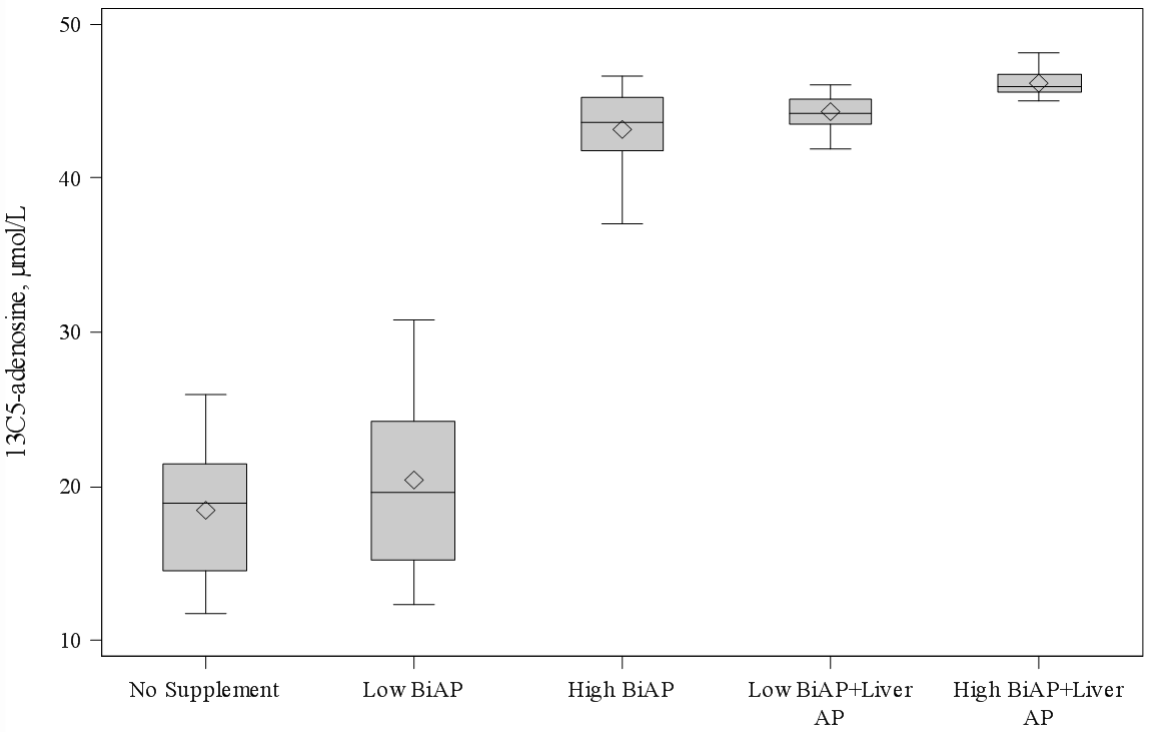
13C5-adenosine production with ex vivo alkaline phosphatase supplementation. ◊ = mean. BiAP = bovine intestinal alkaline phosphatase; AP = alkaline phosphatase. Pairwise comparisons are presented in Table 4.

Results of these assays are shown in Fig 4 and Table 4. Across all groups, AP supplementation resulted in a highly statistically significant increase in 13C5-adenosine production (p<0.0001). Comparison among different AP regimens was also performed, testing for significance at the 0.05 level adjusted for multiple comparisons. Addition of low dose BiAP (500U/L) resulted in a modest increase in 13C5-adenosine of 1.9 μM relative to baseline that did not reach statistical significance. Increasing BiAP to high dose (50,000 U/L) resulted in a statistically significant increase in 13C5-adenosine production (mean increase 24.4 μmol/L). With this level of BiAP activity, ~90% of the 13C5-AMP was converted to 13C5-adenosine. Unexpectedly, use of human liver AP at physiologic levels (500U/L) in addition to BiAP had a dramatic additive effect on 13C5-adenosine production. Addition of physiologic liver AP to low dose BiAP increased adenosine production relative to baseline or low dose BiAP alone (25.5 and 23.6 μmol/L respectively). Furthermore, 13C5-adenosine production with this regimen was comparable to that obtained by the use of supraphysiologic BiAP (44.4 vs 43.7 μmol/L). Addition of physiologic liver AP to high dose BiAP resulted in a smaller but statistically significant increase in 13C5-adenosine production when compared to high dose BiAP alone (2.9 μmol/L).

**Table 4:** Adenosine production with AP supplementation + = significant at the 0.05 level adjusted for multiple comparisons.

Group Comparison	Difference Between Means (μmol/L)	95% Confidence Limits	Significance at 0.05 level
No supplement vs. low dose BiAP	1.9	(-0.8, 4.7)	
No supplement vs. high dose BiAP	24.4	(21.6, 27.2)	+
No supplement vs. low dose BiAP + low dose liver AP	25.5	(22.7, 28.3)	+
No supplement vs. high dose BiAP + low dose liver AP	27.4	(24.6, 30.1)	+
Low dose BiAP vs. high dose BiAP	22.5	(19.7, 25.3)	+
Low dose BiAP vs. low dose BiAP + low dose liver AP	23.6	(20.8, 26.3)	+
Low dose BiAP vs. high dose BiAP + low dose liver AP	25.4	(22.6, 28.2)	+
High dose BiAP vs. low dose BiAP + low dose liver AP	1.1	(-1.7, 3.9)	
High dose BiAP vs. high dose BiAP + low dose liver AP	2.9	(0.1, 5.7)	+
Low dose BiAP + low dose liver AP vs. high dose BiAP + low dose liver AP	1.8	(-0.9, 4.6)	

## Discussion

Relatively little is known about the physiologic role of AP despite conservation of these enzymes from bacteria to man.[35] Missense mutations in the tissue nonspecific form of AP in humans result in failure to hydrolyze PPi and the severe clinical syndrome of hypophosphatasia.[35] Other functions of serum and tissue-based AP are less well understood.

New evidence from in vitro assays and animal models of local and systemic tissue injury points to a possible role for AP as an immune modulator.[22,27–33] Initial work focused on the ability of AP to dephosphorylate lipopolysaccharide and other pathogen associated molecular patterns leading to a decrease in toxicity and immune stimulation.[17,36–37] More recently it has been hypothesized that AP is also capable of converting extracellular adenine nucleotides released during cellular injury (ATP, ADP, and AMP) into adenosine.[33–35] Extracellular adenine nucleotides are pro-inflammatory, cause platelet activation, and may increase tissue injury. Adenosine on the other hand is anti-inflammatory, decreases platelet activation, and may decrease ischemia-reperfusion injury.[24] Adenosine signaling research in humans has primarily focused on the ectonucleotidases CD73 and CD39 with relatively little attention to AP. Recently, though, a novel study by Pettengill *et al* demonstrated the importance of soluble AP to the clearance of exogenous AMP added to plasma from healthy neonates.[26]

To our knowledge the present study is the first to evaluate the role of soluble AP on the clearance of AMP in a disease state. The findings of a strong positive correlation between AP activity and adenosine production as well as a five-fold reduction in adenosine production with selective AP blockade suggest that AP is the primary soluble ectonucleotidase in the setting of infant cardiopulmonary bypass. Also we confirmed significantly lower adenosine production in serum with AP activity ≤80U/L, providing initial biologic plausibility to support our prior clinical findings of worse clinical outcomes in infants with low post-operative AP activity.[20]

Two additional findings regarding the role of native soluble AP in AMP clearance warrant further discussion. First, the relative importance of AP ectonucleotidase activity appears to increase with higher concentrations of AMP. Pettengill *et al* demonstrated relatively little effect of AP blockade on adenosine production when 5μmol/L AMP was provided as substrate in plasma from healthy neonates.[26] When AMP concentrations were increased to 100μmol/L, however, AP inhibition more markedly affected adenosine production. The authors suggest that at physiologic concentrations of AMP, CD73 acts as the dominant soluble ectonucleotidase, with AP taking a more prominent role as AMP increases to levels simulating concentrations found in pathologic states. Our findings support this hypothesis. The correlation between AP activity and adenosine production was higher at higher concentrations of AMP, and the relative contribution of CD73 was lower when more AMP was available as a substrate.

Next, in rewarming serum soluble AP demonstrates a reduced role in the conversion of AMP to adenosine. Part of this diminished role for AP can be directly explained by the marked decrease in AP activity after surgery. Serum AP activity decreases after cardiac surgery with cardiopulmonary bypass in both children and adults.[21,38] Previous work in our group demonstrated a profound decrease in AP activity after infant cardiopulmonary bypass[20] and this finding is again identified in the current study where mean serum AP activity fell 48% from pre-operation to rewarming. Indirect evidence from the study, however, suggests that part of the reduced role for AP in adenosine production may come from an increase in CD73 activity. Limitations on blood volume drawn for research prevented direct measurement of CD73 in this set of patients. However, patients with small decreases in AP activity during rewarming actually demonstrated an increase in adenosine production. This increase was largely eliminated with CD73 inhibition, suggesting that an increase in CD73 might be responsible for these findings. No studies have previously assessed CD73 levels after cardiac surgery, but an increase in CD73 activity would be consistent with previous work on CD73 in other settings involving tissue injury and inflammation.[39,40]

Assessment of native AP activity relative to clearance of AMP prior to separation from bypass is an important finding that may help explain a portion of the variation in the subsequent post-operative course for infants undergoing cardiothoracic surgery.[20] Perhaps more important, our data elucidate a mechanism potentially underlying the beneficial effects seen with preliminary studies of AP replacement therapy in cardiac surgery and other diseases. Animal models of sepsis, inflammatory bowel disease, necrotizing enterocolitis, acute kidney injury, neurologic injury, and myocardial infarction have demonstrated decreased inflammation and reduced tissue injury with local or systemic treatment with bovine intestinal AP.[15,16,18,27–29,41] Multiple mechanisms have been suggested including dephosphorylation of LPS and extracellular adenine nucleotides. Four small phase 2 studies have been completed in adult humans with sepsis, inflammatory bowel disease, and during coronary artery bypass grafting.[22,30–32] All 4 studies showed a positive effect on either inflammation or clinical outcomes, but the mechanism through which AP therapy acts in these diseases remains unclear and none of these studies evaluated the potential role of increased extracellular adenine nucleotide clearance by therapeutic AP.

Our study is the first to evaluate the effect of exogenous AP administration on extracellular adenine nucleotide clearance capacity in human serum. We chose to test AP administration in serum with high concentrations of added 13C5-AMP in order to demonstrate relevance at concentrations representing stressed states.[26] We also tested BiAP and human liver AP in order to evaluate addition of both the proposed therapeutic form and the native form of AP. Both BiAP and human liver AP were able to provide added clearance of 13C5-AMP above that accomplished by the native ectonucleotidases in this model, supporting this effect as a potential mechanism of action of therapeutic AP seen in other studies.[22,30–32] One unexpected finding was the difference in the magnitude of effect with human liver AP compared to BiAP on dephosphorylation of AMP. While the study was not primarily designed to assess different dosing regimens for AP, we had anticipated finding roughly similar potency from BiAP and human liver AP. Instead we identified a roughly 100-fold increased potency for human liver AP compared to BiAP. Human liver AP effectively dephosphorylated AMP at physiologic activity levels, whereas BiAP required supraphysiologic activity levels to have similar effects on AMP. The reasons behind these differences are not clear but may include differences in half-life, species or isoform specific action on AMP, and differential clearance of bovine versus human proteins. Additional studies are needed to determine optimal therapeutic regimens as well as mechanisms behind differential potency of these versions of AP. The recently produced recombinant human AP (hybrid human intestinal/placental AP) designed for therapeutic use should also be evaluated with this mechanism in mind.[18]

### Study Limitations

Our study does have several limitations. Most notably, it is very difficult to assess the effects of AP directly on native extracellular adenine nucleotides and native adenosine production. Once the steady state of production is broken, adenosine is rapidly cleared via ADA1 or taken into cells via ENT1,2 and difficult to measure accurately. As our goal was primarily to understand the role of AP in conversion of AMP to adenosine, we decided to adapt the ex vivo model established by Pettengill, *et* al using exogenous AMP as the supply and trapping adenosine in the serum by removing the cellular content and blocking ADA1 and ENT1,2. Removal of the cellular component removes a source of ectonucleotidases, but allowed us to focus on the serum component that is most relevant from a clinical testing perspective. We also decided to focus only on AMP as a substrate to simplify the model. However, it is likely that AP is capable of dephosphorylating ATP and ADP in this setting and this should be tested in future studies. Our model does not account for tissue level actions of AP and further research is necessary to understand the role of tissue-based AP on organ injury and inflammation in cardiopulmonary bypass. Our study was designed to assess the relationship of serum AP activity and serum capacity to clear AMP. The study does not give significant insight into the etiology of decreased AP activity in this population, nor does it evaluate potential effect modification between perioperative medications and AP dephosphorylation of AMP. Additional studies are ongoing at our center to further evaluate the timing and cause of AP activity loss after infant cardiovascular surgery. Finally, our study does not address the question of what is a clinically important or safe change in serum AMP clearance capacity. While the most common physiology after infant cardiac surgery is one of high systemic vascular resistance and low cardiac output where afterload reduction is the primary therapeutic strategy, overproduction of adenosine may also be a problem leading to excessive vasodilation and hypotension. Overproduction of adenosine may also lead to decreased platelet activity and the potential to increase post-operative bleeding. Future in vivo studies of AP in this setting should address optimal dosing and adenosine production.

## Conclusions

AP represents the primary serum ectonucleotidase after infant cardiac surgery and low post-operative AP activity leads to impaired capacity to clear AMP in this model. The role of AP appears to be particularly prominent with higher AMP concentrations. AP supplementation improves serum clearance of AMP to adenosine, but AP isoenzymes may have differential potency. Together, these findings point to a potential biologic mechanism for AP treatment during cardiac surgery and other states of inflammation/tissue injury.

## Supporting Information

S1 File Detailed Methods Section. (PDF)

S2 File Data files for 13C-AMP to 13C-Adenosine conversion with and without CD73 inhibition. (XLSX)

S3 File Data files for 13C-AMP to 13C-Adenosine conversion with TNAP inhibition. (XLSX)

S4 File Data files for 13C-AMP to 13C-Adenosine conversion with AP supplementation. (XLSX)

## Abbreviations

ADA1: Adenosine deaminase 1
ADP: Adenosine diphosphate
AMP: Adenosine monophosphate
AP: Alkaline Phosphatase
ATP: Adenosine triphosphate
BiAP: Bovine intestinal alkaline phosphatase
CPB: Cardiopulmonary bypass
EHNA: Erythro-9-(2-hydroxy-3-nonyl)adenine
ENT 1,2: Equilibrative nucleoside transporter 1 and 2
TNAP: Tissue non-specific alkaline phosphatase
