# Supplementary material for: Alkaline Phosphatase, Soluble Extracellular Adenine Nucleotides, and Adenosine Production after Infant Cardiopulmonary Bypass
Source: PLoS One. 2016 Jul 6;11(7):e0158981. doi: 10.1371/journal.pone.0158981 (PMC4934870; doi:10.1371/journal.pone.0158981)
Supplement: S1 File — (PDF) [file pone.0158981.s001.pdf]

Detailed Methods:

As aforementioned, low (5 µmol/L; patients 1-25) and high (50 µmol/L; patients 26-50) dose 13C5-AMP were used to simulate normal/stress concentrations of AMP.[1] In all assays, adenosine deaminase 1 (ADA1) and equilibrative nucleoside transporter 1 and 2 (ENT1,2) were blocked with erythro-9-(2-hydroxy-3-nonyl)adenine (EHNA, 3mM) and dipyridamole (400 µM) respectively. Reactions were terminated after 15 minutes by addition of five volumes acetonitrile/ methanol containing 5 µmol/L d1-adenosine as internal standard [Tocris Cookson; Bristol, UK]. Samples were centrifuged at 13,000g for 10 minutes at 4°C and transferred into HLPC vials for the same-day analysis.

Liquid chromatography – tandem mass spectrometry (LC/LC-MS/MS): To assess the conversion of exogenous 13C5-AMP to 13C5-adenosine in patient serum, we employed a modification of a previously described assay.[2,3] A Dionex Ultimate 3000 HPLC (Thermo Fisher Scientific, Sunnyvale, CA) coupled to an API QTRAP 5500 mass spectrometer (AB Sciex, Concord, ON) equipped with an electrospray ionization (ESI) source (in negative ionization mode) was employed for quantitation of adenosine and AMP. Following mass transitions (m/z) were monitored: 346.3→79.0 (native AMP), 351.2→134.1 (13C5-AMP), 266.2→79.0 (native adenosine), 271.2→134.1 (13C5-adenosine), and 267.1→135.2 (adenosine-d1, internal standard). The ESI interface was set to 500 °C with ion spray voltage at -4500 V. The nitrogen curtain gas was at 20 L/min.

100 µl of sample extract were loaded onto the C18 cartridge desalting column (50 × 4.6 mm Zorbax C18, 5 µm particle size, Agilent Technologies) and were washed with 1 mL/min of 95% 4 mmol/L dibutylammonium formate (DBAF) buffer and 5% methanol for 0.7 min. Thereafter, the switching valve was activated and the analytes were back-flushed from the desalting column onto the analytical column (250 × 4.6 mm Phenomenex Synergy Hydro C18, 3 µm particle size, Phenomenex, Torrance, CA, USA). The nucleotide peak separation was performed using the 2 mmol/L DBAF buffer (as solvent A) and methanol (as solvent B) with the following gradient: from 95% to 50% solvent A within the first 2 min, from 50% to 2% solvent A from 2-4 minutes, after which the gradient was kept at 2% solvent A for additional 4 minutes. The following 2 min were used to re-equilibrate the analytical column to the starting conditions. The flow rate was 0.5 mL/min, and the column temperature was maintained at 35 °C. Adenosine and AMP were quantified by calculating the under-the-peak ratios of analyte to internal standard and comparing with those of included calibration curves for both analytes, respectively. The calibrators ranged between 0.025 µM to 50 µM for 13C5-AMP and 13C5-adenosine, respectively.

Supplemental References:

1. Pettengill M, Robson S, Tresenriter M, Millan JL, Usheva A, Bingham T, Belderbos M, Bergelson I, Burl S, Kampmann B Gelinas L, Kollmann T, Bont L, Levy O. Soluble ecto-5'-nucleotidase, alkaline phosphatase, and adenosine deaminase activities in neonatal blood favor elevated extracellular adenosine. *J Biol Chem 2013*; 288(38):27315-26.

2. Klawitter J, Schmitz V, Klawitter J, Leibfritz D, Christians U: Development and validation of an assay for the quantification of 11 nucleotides using LC/LC-electrospray ionization-MS. *Anal Biochem,* 365**:** 230-239, 2007

3. Henry CJ, Casas-Selves M, Kim J, Zaberezhnyy V, Aghili L, Daniel AE, Jimenez L, Azam T, McNamee EN, Clambey ET, Klawitter J, Serkova NJ, Tan AC, Dinarello CA, DeGregori J: Aging-associated inflammation promotes selection for adaptive oncogenic events in B cell progenitors. *J Clin Invest,* 125**:** 4666-4680, 2015
